# Bayesian Forecasting of Extreme Values in an Exchangeable Sequence

**DOI:** 10.6028/jres.099.050

**Published:** 1994

**Authors:** Bruce M. Hill

**Affiliations:** Department of Statistics, University of Michigan, Ann Arbor, MI 48109

**Keywords:** Bayesian forecasting, exchangeability, long-tailed distributions, record values, tail-index estimator

## Abstract

This article develops new theory and methodology for the forecasting of extreme and/or record values in an exchangeable sequence of random variables. The Hill tail index estimator for long-tailed distributions is modified so as to be appropriate for prediction of future variables. Some basic issues regarding the use of finite, versus infinite idealized models, are discussed. It is shown that the standard idealized long-tailed model with tail index α ≤ 2 can lead to unrealistic predictions if the observable data is assumed to be unbounded. However, if the model is instead viewed as valid only for some appropriate finite domain, then it is compatible with, and leads to sharper versions of, sensible methods for prediction. In particular, the prediction of the next record value is then at most a few multiples of the current record. It is argued that there is no more reason to eschew posterior expectations for forecasting in the context of long-tailed distributions than to do so in any other context, such as in the many applications where expectations are routinely used for scientific inference and decision-making. Computer simulations are used to demonstrate the effectiveness of the methodology, and its use in forecasting is illustrated.

## 1. Introduction

Consider a sequence *X*_1_,…,*X_n_* of positive random variables that is exchangeable. We say that *X_n+_*_1_ is a (new) record value if *X_n_*_+1_*> X_i_*, for *i* = 1,…,*n*. See [2] for some related discussion of record values in the iid case. The problem that we address concerns forecasting of the next observation, *X_n_*_+1_, given that it is a record value, conditional upon the data *X_i_ = x_i_*, for *i* = 1,*…*,*n.* In other words, given that *X*_*n*+1_ sets a new record, how large will it be?

In the Bayesian approach, with squared error loss, the forecast of X*_n_*_+1_, conditional upon the data *X*_1_,…,*X_n_*, and upon *X_n_*_+1_*>max* [*X*_1_,…,*X_n_*], is simply the posterior expectation of *X_n_*_+1_ conditional upon the same information. Note that if a sequence is exchangeable, then the future variables are also conditionally exchangeable, given the realization of the first *n* variables. Hence each of the next *N* observations has in fact the same posterior predictive distribution. The posterior expectation for *X_n+j_*, conditional upon *X_n+j_* being larger than each of the first *n* observations, is then the same for each *j* ≥ 1. It may be noted that there are two quite different questions that arise concerning the forecasting of future record values. The first concerns the forecasting of when the next record value will occur, while the second concerns the forecasting of the *magnitude* of the next record value. In this article we only consider the second question.^1^

Although we focus attention here only on the prediction of the magnitude of *X_n_*_+1_ given that it sets a new record, there is a relatively straight-forward extension of these results to the evaluation of the posterior expectation of *X_n+j_*, given that it sets a new record. To obtain the prediction of the next record value, conditional upon the data *x*_1_,…,*x_n_*, and upon *X_n+j_* being the next new record value, we must evaluate the posterior expectation of *X_n+j_*, conditional upon the collection of inequalities that define the event that *X_n+j_* is the next record value. This can be done by a generalization of the procedure for forecasting *X_n_*_+1_ conditional upon its being the next record value. For example, the posterior expectation of *X_n_*_+2_, conditional upon its setting a new record, can be obtained by conditioning upon the event that *X_n_*_+1_ sets a new record, and then making the same type of evaluation as above for *X_n_*_+1_, given that it is a record value; or alternatively, by conditioning upon the event that *X_n_*_+1_*< max*[*X*_1_,*…X_n_*], and then evaluating the posterior expectation of *X*_*n*+2_, given that it is larger than *max*[*X*_1_,*…,X_n_*]. Since in the Bayesian framework with a specified *a priori* distribution, the posterior probability that *X_n_*_+1_ sets a new record is known, there is no difficulty in principle in extending the analysis for the posterior expectation of *X_n_*_+1,_ given that it sets a new record, to the forecasting of the magnitude of future record values. Explicit algorithms for doing so will appear in a later paper.

Although the present paper deals only with the evaluation of the posterior expectation of *X_n_*_+1_, given that it sets a record, we shall nonetheless sometimes speak of forecasting the magnitude of future record values, since this can be achieved by the same basic methods. Similarly, one can obtain the posterior expectation of the maximum over some finite horizon, say the maximum of *X_n_*_+1_,*…,X_n+N_*, given that this maximum exceeds our current record value. This is a problem of considerable practical importance both in economic forecasting of interest rates, and in engineering design, where for example, one desires to build a structure capable or withstanding severe winds or earthquake tremors over a certain period of time. To the best of my knowledge such forecasting has never been attempted before in the sense of providing a procedure that could be recommended for serious consideration in real-world problems.

If we assume a conventional statistical model with some unknown parameter *0*, then in principle these are straight-forward Bayesian problems, since one can integrate out unknown parameters with respect to their posterior distribution, to obtain the predictive distribution for a new observation; and then condition also upon such a new observation being a record value, in order to answer the question. For example, one could obtain the posterior expectation and variance for *X_n_*_+1_, given that it is a record value. However in typical real-world problems involving forecasting of such extreme values, the model is always uncertain and often unreliable. This is especially so in the tails of the distribution, where there is little, if any, past data to rely upon. Thus to obtain reliable forecasts requires serious attention to model uncertainly. See Hill [7] for discussion of the selection of models from a Bayesian viewpoint, Poirier [8] for a Bayesian analysis of some theoretical models in economics, and Singpurwalla and Meinhold [9] for Bayesian robustification theory in a closely related area.

In this paper we attempt to deal with the problem by using the formulation for inference about the tails of the distribution initiated in [1], See [10] for an exposition, and Csörgö et al. [11] for related asymptotic theory. This approach utilizes only the upper order statistics of the past data for inference about the upper tail, since it is only such order statistics that fall in the upper tail where the form of the distribution is assumed known. Seriously to utilize the information in the other order statistics requires knowledge concerning the global form of the distribution, and such knowledge is often unavailable. Suppose that given the parameter a, the upper tail of a distribution *F* on the positive real line is of algebraic form, with tail index *α.* We assume that

$$1-F\left(t\right)=P\left(X>t|\alpha \right)≃C\times l^{-\alpha },$$

for *C*>0, *α*>0, and *t* in some interval (*A, k*) that is considered relevant for prediction of future observations. It is supposed that a random sample *X_i_ = x*_1_, for *i* = 1,…*n*, from the distribution is available, and based upon this data we wish to forecast the next observation *X_n_*_+1_. Such prediction in the Bayesian context amounts to putting forth a posterior distribution for *X_n_*_+1_, that is obtained by integrating out unknown parameters such as *α*, with respect to their posterior distribution, and then making appropriate forecasts by minimizing posterior expected loss with respect to some loss function. In this article we consider only squared error loss, but our methods can be used in connection with any loss function believed appropriate. See Aitchision and Dunsmore [12] and Maret [13] for the Bayesian theory and methodology of such predictive distributions.

Often a simple summary of the posterior predictive distribution, such as the posterior expectation and variance of X*_n_*_+1_, suffices for many practical purposes. In typical applications *A* will be the largest order statistic of the past data, *k* can sometimes be + ∞, but for reasons discussed below will often instead be some modest multiple of *A*. We might be interested, for example, in forecasting the next observation, *X_n_*_+1_, conditional upon its being between *x*^(1)^ and 5*× x*^(1)^, where *x*_(1)_ is the largest order statistic of the past data. Forecasting of such a record value is an especially difficult part of the overall forecasting problem, since by assumption there is no past data of this magnitude. Yet in forecasting extreme values, it is necessary to consider precisely the situation in which the observation is more extreme than anything yet seen. For example, in designing a structure to resist high winds, one must make allowance for forces more extreme than have yet been experienced. It would be foolish to imagine that such forces have already been observed at their maximum.

The best that one can do in such circumstances is to use what relevant theory exists, making sure that such theory is compatible with the data that has been seen. In this article we shall rely on the theory of long-tailed distributions, in which the tail is known to be of algebraic form at least in some interval. Many data sets are known to be of this form. Examples include income distributions, city size distributions, distributions of genera by species, insurance claim sizes, word frequency distributions, stock market fluctuations, and many others. See Zipf [14] for graphical presentation of a great variety of data in support of his theory for long-tailed distributions. Several theoretical models have been proposed for such data. These include the probability models of Yule [15], Hill [16,17,18], Hill and Woodroofe [19,20], and Hill, Lane and Sudderth [3,4]. See Johnson and Kotz [21] for discussion of the model of Hill [22,17], which was the starting point for the later models. As pointed out by Chatterjee and Yilmaz [23], some of these models are related to stochastic models for chaos.

We are particularly interested in the case where *α* is not large, so we are dealing with a truly long-tailed distribution. For any *α* >0 the distribution of *X_n_*_+1_ is proper, even when *k = ∞.* However, for fixed known *α ≤* 1 the expectation of *X_n_*_+1_ is infinite if there is no finite upper bound for the data, and the variance of *X_n_*_+1_ is infinite if α ≤ 2. Also, if α ≥ l is unknown, which is ordinarily the case, the posterior distribution for *α* must give sufficiently small weight to values of *α* near 1, in order for the posterior expectation of *X_n_*_+1_ to be finite. This gives rise to an important practical issue for Bayesians, since the predictions are then very sensitive to the precise form of the *a priori* distribution for *α* near 1, and the results are not robust. Similarly, if *α* ≥ 2 is unknown, the posterior distribution for *α* must give sufficiently small weight to values of *α* near 2, in order for the posterior variance of X*_n_*_+1_ to be finite.^2^

In view of such nonrobustness, it is necessary to proceed more carefully than in most problems of statistical inference and prediction. Our method is to take explicit account of the boundedness of the observations. In many real world applications of extreme value theory, where one deals with maximal temperatures, wind velocities, rain fall, etc., the data are generally considered to be bounded. For example, a wind velocity even double the highest ever previously experienced, must be regarded as extremely improbable. Even if such could occur, it might be regarded as indicating a basic change in climate such as would invalidate all standard assumptions, and so require modification of existing theory. This suggests that a realistic analysis of the problem should incorporate a finite upper bound, *say K*, for the data.^3^ Such a bound might be taken a good deal larger than is ordinarily believed reasonable. A 10-fold increase above a previous record value that was based upon substantial data would often be too large, but is worthy of consideration. If such an upper bound is incorporated in the analysis, then as shown below, even if *α* ≤ 1 there is no problem with infinite moments. We will typically assume some known finite upper bound *K*, perhaps much too large, but we will not necessarily assume that a ≥ 1, and will let the data speak for themselves in this regard. Since the density in the tail is proportional to *t*^−^*^α^*^−1^, we see that *α* = 0 corresponds (in the tail) to a uniform distribution for the logarithm of the observation. Such a distribution is often used by Bayesians to represent diffuse *a priori* knowledge about a positive quantity such as a variance.

Our precise model is as follows. We assume that there exists a known constant *K* such that 0 *≤ X ≤ K*, so that *K* is a known upper bound for the data. In applications, ordinarily *K* < ∞, but for completeness we shall also discuss the case *K* = ∞, which is sometimes appropriate and is mathematically convenient when *α* >2 + *∊*>2, in which case no problems arise due to infinite first or second moments. We do not assume in applications that one can necessarily determine a smallest such *K*, but merely that one can pick some bound. We also assume that there exist constants *k* and *A* with *K > k > A* > 0, such that the tail is algebraic, to an adequate approximation, for *A* ≤ *t* ≤ *k*, with 0 mass beyond *K.* Let *x*_(1)_>…>x_(_*_n_*_)_ be the descending order statistics of the past data. Ordinarily we take *A* to be the largest order statistic of the past data, *A* = *X*_(1)_. The quantity *k* is the key variable in our analysis. It represents the point up to which the algebraic assumption is assumed to be valid, *k* is not a parameter in the usual sense, but is more in the nature of a decision variable, since in applications the tail will not be *exactly* algebraic in any interval, but it will nevertheless be reasonable to act as if it were approximately of this form for some intervals. The selection of *k* in part acts as a means to specify the portion of the distribution that we are particularly interested in. Even if *X > k* we may not be interested in forecasting *X* for such extreme values, since the occurrence of such would force us to reconsider our modelling assumptions, as in [7,24,25].

We are in effect assuming a model in which the algebraic behavior holds, given α, to a satisfactory approximation for *A ≤ X ≤ k*, and that eventually there is 0 (or negligible) mass beyond some known *K > k.* We assume that the same *k* is appropriate for all values of *α* being given positive weight. Between *k* and *K* there must be a transition from the algebraic tail behavior up to *k* and the negligible mass beyond *K.* In this transition zone the tail of the distribution may not even be approximately algebraic, and if algebraic, may have a different tail index. The mass between *k* and *K* need not be entirely negligible, but we assume there is no data-based or other information concerning the form of the mass distribution in this interval, apart from the fact that the total mass in the interval is smaller then *C × k^−α^*, as is required by the model. If *k* is large enough, then *C × k^−^*^α^, although not entirely negligible, may be sufficiently small so that the mass between *k* and *K* < ∞ has only a slight effect upon the posterior moments for *X_n_*_+1_, We shall assume that this is the case, so that the tail distribution is of algebraic form from *A* to *k*, while beyond *k*, although not 0 or entirely negligible, the mass is of no practical importance for the assessment of the posterior moments of *X_N_*_+ 1_.

Typically, the posterior expectation of $C\times x_{\left(1\right)}^{-\alpha }$ will be of order of magnitude 1/(*n* + 1) based on a previous sample of size *n.* Compare the maximum-likelihood estimator *Ĉ*_1_ of [1, p. 1168]. This also corresponds to the fiducial analysis of Fisher [26, p. 210], and to the Bayesian non-parametric procedure *A_n_* of Hill [22,27,35]. Thus before observing *X*_1_,…,*X_n_*, because of the exchangeability there is an unconditional probability of 1/(*n* + 1) that *X_α_*_+1_ will be the maximum, which suggests that even conditionally this will often be of the right order of magnitude. As shown in [5], there is an explicit parametric model, called a splitting process, for which this evaluation holds exactly, and such an evaluation is coherent in the sense of de Finetti [28,29].

The constant *K* plays virtually no direct role in the following analysis, but is important because of the delicate issues that arise when *α ≤* 2. In this case if there were no finite upper bound *K* and the algebraic tail were assumed valid everywhere beyond *A*_1_ then the posterior predictive variance of the next observation would be infinite; and the predictive expectation would also be infinite unless the *a priori* distribution for *α* gave sufficiently small weight to values near 1. There is no known reason that *a* must be larger than 2, or even larger than 1, and the data may in fact clearly suggest that it is smaller than 1. But an infinite predictive expectation would not correspond to any real world problem that I know of concerning extreme data, and I doubt that one could seriously recommend such predictions. For example, they would lead to terribly poor performance if predictions were made and assessed according to some proper scoring rule or loss function. This change in viewpoint to reflect the boundedness of the data gives rise to some surprising consequences with regard to prediction.

The key choice concerns not *K* but *k*, since even if there were a known finite upper bound *K* for the data, it might still not be appropriate to assume the algebraic form all the way up to *K*, but only that in the domain of practical importance the tail is of this form, say up to *k*, which is equal to some appropriate upper percentile of the distribution. This is in essence a modelling assumption, just as when we assume that the normal model for data is sufficiently closely satisfied to be useful in the analysis of that data. Modelling assumptions are rarely exactly true, but they are sometimes indispensable in order to proceed, and often give useful results. See [7,25,27]. The form of analysis that we recommend is a conditional analysis, given a specification of *k.* For example, with *A* = *x*_(1)_, we consider predictive inference about the next observation given that it lies between *x*_(1)_ and some *k > x*_(1)_. If *L = k/x*_(t)_, then we find that it typically makes a great difference whether *L* is of order 5 or order 100, both with respect to the posterior predictive mean and the posterior predictive variance for the next observation. Based upon the mathematical and computer analysis in the next sections, we recommend that the forecaster make a choice of *L*, usually with *L* ≤ 10 and sometimes even with *L* = 2. To illustrate, when *L* is chosen to be 3, the adequacy of our modelling assumption depends on whether it is or is not the case that the algebraic form holds between *x*_(1)_ and 3×*x*_(1)_, with the mass beyond 3×*x*_(1)_ no longer even approximately of the algebraic form with the same *α* as between *x*_(1)_, and 3 ×*x*_(1)_, and also with the mass beyond 3 ×*x*_(1)_ sufficiently small so that for practical purposes it can be ignored. In principle the optimal choice of *k* is the largest value for which the algebraic assumption holds exactly (or in a suitable sense, approximately); while beyond that *k* the tail is no longer of that same form, and also is of little practical importance in the evaluation of the first two posterior predictive moments. It would be difficult if not impossible in typical real-world problems to find such an optimal *k*, and so we recommend that several values of *k* be chosen, yielding different values for the posterior predictive moments, and then by means of judgment and data-analytic methods that a choice be made to yield a forecast. See for example Sec. 5 of [1] for a closely related type of data-analysis. Such analyses must be made on a computer, rather than purely mathematically, and can be quite demanding computationally.

We emphasize that it does not seem possible to avoid such considerations as to the choice of *k*, since in even the best of cases, where the tail of the distribution is known to be of the algebraic form in the domain of interest, the only alternative to such an analysis is to simply ignore the boundedness of the data, and take *k* = ∞, But then our prediction of the next record value can become infinite, which is absurd in most real-world problems. Hence the algebraic tailed model with 1 ≤ *α* ≤ 2 is not compatible with unbounded data unless the *α priori* distribution is chosen to give suitably small weight to values of *α* close to 1. There may be little or no evidence for choosing the *a priori* distribution in this way, and it does not seem appropriate to do so merely to avoid the issue, just as it does not seem appropriate to replace the expectation by the median merely to avoid the issue. At any rate, this article shows that effective predictions can be made with any prior distribution for *α*, including cases where *α* ≤ 1, provided that one can justify some finite upper bound *K* for the observations.

Our underlying motivation is that given the unreliability of assessments of the far upper tail of a distribution, for predictive purposes it may be appropriate to ignore this far upper tail, i.e., the part beyond *k*, or cquivalently, to condition upon *X* falling in some finite interval, say (x_(1)_,*L* × *x*_(1)_), for which the algebraic assumption is believed to be valid, and beyond which there is no assumption that is believed trustworthy. It is implicit in this analysis that there is little mass beyond *k*, and that in ignoring the case *X ≥ k* for some appropriately chosen *k*, one loses little, while gaining the power of a statistical analysis based upon the extreme value model with some α > (). In the case of a known finite upper bound *K*, in effect we perform conditional inference, given that the observation is not too large, and then examine sensitivity to the choice of *k.* The same is true if the random variable is unbounded and *K* = *∞*, since again beyond a certain percentile one would have no empirical basis for any assumption in the far upper tail. Whatever extreme value theory exists for tails of distributions could not be expected to hold literally in the far upper tail of the distribution, where no data has been observed. Nevertheless, one may have to make some forecasts, and it would appear reasonable to assume that the algebraic assumption holds for at least some distance beyond *x*_(1)_. ff this, or some other assumed model does not hold beyond *x*_(1)_ then plainly no serious theory-based forecasting is possible. But if through data analysis, as in [1,26], it has been discovered that the algebraic assumption is acceptable for say the upper *r* + 1 order statistics of the past data, then it would be reasonable to anticipate that this will also be true for some distance beyond *x*_(1)_. A Baycsian theory of data analysis is put forth in [25] which indicates how the classical Bayesian approach must be modified to deal with issues that arise from such data analysis.

Finally, real world data sets of interest in regard to the forecasting of extreme values are not necessarily of the long-tailed algebraic form that we have discussed. In this case we recommend that a transformation be first applied to the data in order to make the upper tail of the long-tailed form. For example, if the tail is of Weibull form, then the transformation to exp *X*^1^ yields an algebraic tail, as discussed in [1,10]. When the form of the tail is unknown, data-analytic methods can be used to determine an appropriate transformation. In this way, having learned how to forecast extreme tails for the long-tailed distributions as a type of standard case, we can also apply our methods to distributions not of this form in the upper tail, and then take the inverse transformation to forecast the extreme values in the original units in which the data were measured. Such methods are quite common in statistics, for example in transforming data in order to obtain approximate normality, using normal methods for analysis of the data, and then transforming back to the original units. In the Baycsian scenario it is even possible to provide a strong justification for these methods, since conditional upon the data, one can quite freely transform the parameters, and obtain the posterior distribution for the new parameters by the usual calculus of transformations.

## 2. Predictive Moments for Known *α*

Our object is to evaluate, as meaningfully and robustly as possible, the posterior moments

$$E\left(X_{n\;+\;1}^{1}|A⩽\;X_{n+1}⩽\;k\right),$$

for specified *A* and *k*, and *i* = 1,2. The primary application will be in the case where there has been a previous sample, *X_t_,…,X_n_.* Let *D* denote the data *X*_1_ *= x*_1_,…,*X_n_ = x_n_.* Given this data, we wish to forecast the next observation *X_n_*_+1_. It is notationally convenient to refer to *X_n_*_+1_ as *X* from now on. Since *A* will usually be held fixed, we suppress it in the notation. To evaluate the posterior predictive expectation of *X* we first condition on α, to obtain

f(k,α)=E(X|A⩽X⩽k,α),

and then we take the expectation of this quantity with respect to the posterior distribution of *α* to obtain the predictive expectation of primary interest.

Based upon our assumption that the tail is algebraic between *A* and *k*, we obtain

$$f\left(k,a\right)=\frac{\int_{1}^{k}x^{-n}\;\mathrm{dx}}{\int_{1}^{k}\;x^{-a-1}\;\mathrm{dx}}.$$

For $L=\frac{k}{A}$, this yields:

$$f\left(k,\alpha \right)=\{\begin{array}{cc} A\times \frac{\alpha }{n-1}\times \frac{1-L^{1-m}}{1-L^{-n}} & \text{if}\;\alpha \neq 0,1\\ A\times \mathrm{In}\left(L\right)\times \frac{1}{L-1} & \text{if}\;\alpha =1\\ \frac{A\times \left(L-1\right)}{\mathrm{In}\left(L\right)} & \text{if}\;\alpha =0. \end{array}$$ (1)

For α ≠0,1, we can also write:

$$f\left(k,\alpha \right)=A\times \frac{\alpha }{\alpha -1}\times L\times \frac{L^{\alpha -1}-1}{L^{\alpha }-1}.$$ (2)

A similar equation is available for *f^(2)^(k, α) ≡ E(X^2^|A≤X≤K, α)*. We obtain:

$$f^{\left(2\right)}\left(k,\alpha \right)=\{\begin{array}{cc} A^{2}\times \frac{\alpha }{\alpha -2}\times \frac{1-L^{2-\alpha }}{1-L^{-\alpha }} & f\;\alpha \neq 0,2\\ 2\times A^{2}\times \ln\left(L\right)\times \left[\frac{L^{2}}{L^{2}-1}\right] & \text{if}\;\alpha =2\\ A^{2}\times \frac{L^{2}-l)}{2\times \ln\left(L\right)} & \text{if}\;\alpha =0. \end{array}$$ (3)

The posterior predictive variance for a future record value *X*, given *α*, is therefore

$$V(k,\alpha )\equiv f^{\left(2\right)}\left(k,\alpha \right)-{\left[f\left(k,\alpha \right)\right]}^{2}.$$ (4)

It follows from (2) that for *α* > 1, as *L* → ∞ we have

$$f\left(k,\alpha \right)∼A\times \frac{\alpha }{\alpha -1}.$$ (5)

When *α* > 1, the right-hand side of (5) decreases from ∞ for *α* = 1 to the value 2 × *A* when *α* =2, with the value 3 × *A* when *α* = 1.5. Provided that *α* is bounded away from 1 this expectation remains bounded.

For *α ≤* 2, the posterior predictive variance goes to ∞ as *L*→∞. If we define ∊ =2 − α >0 then for large *L*

$$f^{\left(2\right)}\left(k,\alpha \right)\approx A^{2}\times \alpha \times \frac{L^{e}-1}{\epsilon }.$$ (6)

For each *L*>1, and for *∊*>0, the function $\phi \left(\varepsilon \right)=\frac{L^{a-1}}{\epsilon }$ is monotonically increasing in ∊. For 0 < ∊ ≤ 2 it has a maximum value of $\frac{\left[L^{2}-1\right]}{2}$ when ∊ = 2, and an infimum of In(*L*) as *∊*→0. For large *L*, as *∊*→0 we see from Eqs. (3), (5), and (6), that

$$V\left(k,\alpha \right)\approx A^{2}\times (\alpha \;\ln\left(L\right)-{\left[\frac{\alpha }{\alpha -1}\right]}^{2}).$$ (7)

From Eq. (3), it follows that for *α* >2 the posterior predictive variance remains bounded, and as *L*→∞ tends to the limiting value

$$A^{2}\times \frac{\alpha }{\left(\alpha -2\right){\left(\alpha -1\right)}^{2}}.$$ (8)

Now consider the forecasting of the maximum of *N* future observations, Define

$$M=max\left[X_{n\;+\;1,\ldots ,}X_{n+N}\right],$$

and let π*(*α*,*C*) be the posterior distribution for *α*,*C*, based upon the data *D.* The likelihood function *L*_2_(*a*,*β*) of [1], when converted from lower tail to upper tail inference, can be used to obtain this posterior, distribution. For *t >A*, we have

$$\begin{array}{c} P\left(M>t|D\right)=\int_{0}^{\infty }\int_{0}^{\infty }\left[1-(1-C\times t^{-\alpha {)}^{N}}\right]\\ \pi *\left(\alpha ,C\right)d\alpha \;dC. \end{array}$$ (9)

When *N* = 1this gives the posterior predictive distribution for a single new observation considered earlier, except that here we have not yet conditioned upon *X ≥ A.* Just as before, one can consider the posterior moments of *M*, given that *M* ≥ *A*. When *N* is not small it is very probable that *M* ≥ *x*_(1)_> so that a new record will be set. Thus for large *N* the predictive distribution of M will be approximately the same as the predictive distribution of *M*, given *M* ≥ *x*_(1)_.

In Table 1 we present for several values of *α* the predictive moments as obtained by numerical integration. The predictive mean is denoted by E*(X) and the predictive standard deviation by SD*(X). The column labelled DIST gives the posterior predictive probability that *X* is larger than 2, 3, and 5 times *A*. Values of *α* go from .10 to 1.90, and values for *L* go from 1.25 to 10^5^. It can be checked that the above asymptotic formulas hold quite closely for fixed *α.*

We see from Table 1 that the posterior expectation of *X*, given that *X > A*, is only a few multiples of *A*, even when *α* is as small as .10, provided that *L* ≤ 10. in an important class of application *A* is taken to be *x*_(1)_, so that the real action takes place with regard to a few multiples of the largest observation yet observed. When *L* ≤ 2 we see that the value of *α* between .10 and 1.90 has very little effect on the posterior predictive first and second moments. On the other hand, when *L* is very large the value of *α* has a huge effect. For example, the posterior expectation drops from 37,297 × *A* when *L* = 10^6^ to 2.11 *× A*, as *α* changes from .10 to 1.90. The choice of *L* can make a huge difference when *α ≤* 1 However, in many applications of extreme value theory, it could safely be assumed that *L ≤* 10, in which case *L* has only a minor effect even when *α*. The choice of *L* has a greater effect with regard to the predictive variance, but again if *L ≤* 10 there is substantial robustness.^4^ Thus the first conclusion that we draw is that in a real-world problem, where there has been substantia] data, such as with regard to wind velocities, temperatures, etc., and where one does not take seriously the possibility of the next record value being an enormous multiple of the current maximum, the precise choice of *α* and *L* has a limited effect upon the forecast. This is precisely what we are aiming for, namely an approach in which one can seriously input *α priori* knowledge regarding *α* and *L* in such a way as to sec clearly the real but limited effect of such choices.

Table 1 refers to the case of known a. In practice *α* will ordinarily be unknown. The Bayesian approach is to employ some *α priori* distribution π for *α*, obtain the posterior distribution for *α* given *D*, and then obtain the posterior expectation of *X*, given that *A ≤ X ≤ k.* For a specified *k*, this posterior expectation can be written as

f(k)=E[E(X|D,A⩽X⩽k,α)]=E[f(k,α)], (10)

where the last expectation is taken with respect to the posterior distribution of *α.* Similarly, the posterior second moment for *X* is obtained by evaluating

$$f^{\left(2\right)}\left(k\right)=E\left[E\left(X^{2}|D,A⩽X⩽k,\alpha \right)\right]=E\left[f^{\left(2\right)}\left(k,a\right)\right].$$ (11)

We employ the theory of [1] to obtain a likelihood function for the parameter *α* based upon the upper order statistics of the past data. We first condition upon the upper *r*+1 order statistics of the data lying in the region where the tail is of algebraic form, i.e., larger than *D* of [1], and then condition upon the values of the ratios of upper order statistics *v_i_* =*x*^(^*^i^*^),^/*x*^(^*^i^* ^+ 1)^ for *i* = 1,…,*r.* As shown in [1], if we are indeed in the upper tail of the distribution where the algebraic forni holds, then conditional upon *α*, the quantities *e*_1_ = i ×1n *v*, arc independent with a common exponential distribution having parameter *α.* A sufficient statistic for *α*, conditional upon the *v_i_* and *r*, is then

$$t=t(r)=\sum_{i=1}^{r}e_{l}.$$ (12)

The (conditional) likelihood function based upon *r* and *t* is then

$$L\left(\alpha \right)\infty \alpha^{r}\times \exp\left[-\alpha \cdot t\right],$$ (13)

for *α* >0. In conjunction with some *α prion* distribution for *α* this likelihood function can be used to obtain the posterior distribution for *α*. If *k* is large and *a* > 1, we see from (5) that

$$E\left(X|\;D,A⩽X⩽k,\alpha \right)\approx A\times \frac{\alpha }{\alpha -1}.$$ (14)

In general, the predictive moments of *X* can only be obtained by numerical integration. In Sec. 4 we examine the sensitivity of such quantities to the data, choice of *L*, and choice of *a priori* distribution for α. The case *k* = ∞, however, has a closed form analytic solution for a Gamma *α priori* distribution of *α*, and this contributes some insight into the behavior or the posterior moments of *X.*

## 3. *k* = ∞

In this section we examine the special case in which the distribution is known to be algebraic everywhere beyond *A.* In this case, in order for posterior moments to be finite, we will have to assume that *α* is sufficiently large. It follows from Eq. (1) that the posterior expectation of *X_α_*_,1_, given that it is in the upper tail and *α*, is finite if and only if *α* > 1. In the Bayesian analysis, with an *a priori* distribution for *α*, the unconditional posterior expectation of *X* is finite if and only if the *α priori* distribution sufficiently downweights values of *α* near 1.

We can gain some insight by supposing that *a* > 1 has the prior distribution

$$\pi \left(\alpha \right)=c\times {\left(\alpha -1\right)}^{\delta -1}\exp\left[-\beta \left(\alpha -1\right)\right],$$

for *δ, β* > 0, where *c* = Γ(*δ*)/*β^δ^* is a proportionality constant. In other words, we give *α* − 1 >0 a Gamma *a priori* distribution. If 5 > 1 we obtain from Eq. (1) that the posterior expectation of *X/A*, given *X* ≥ *A*, is

$$\begin{array}{c} E\left(\frac{\alpha }{\alpha -1}|D\right)\\ =\frac{\int_{0}^{\infty }{\left(1+s\right)}^{r+1}\times s^{\delta -2}\times \exp\left[-\left(t+\beta \right)s\right]ds}{\int_{0}^{\infty }{\left(1+s\right)}^{r}\times s^{\delta -1}\times \exp\left[-\left(t+\beta \right)s\right]ds}. \end{array}$$ (15)

This expectation is finite provided that *δ* > 1.

For positive integral values of *r* we can expand the powers of 1 *+s* using the binomial theorem, and this allows us to make explicit evaluations. To illustrate, if *r* = 1 as in the forecasting of city sizes in Tables 6 and 7, we have

$$\begin{array}{c} E\left(\frac{\alpha }{\alpha -1}|D\right)=\frac{1+2\left(\delta -1\right)/\left(t+\beta \right)+\delta \left(\delta -1\right)/{\left(t+\beta \right)}^{2}}{1+\delta /\left(t+\beta \right)}\\ \times \frac{t+\beta }{\delta -1}. \end{array}$$ (16)

This reveals the manner in which the expectation blows up as *δ* →1. When *δ* = 2, the right-hand side can be written as

$$1+\frac{\left(t+\beta \right)\left(t+\beta +1\right)}{t+\beta +2}.$$

For *i* + *β* = 1, we obtain the value 1.67. This is comparable with the values in Tables 2, 3, and 4, when *r = t* = 1, and L ≤ 5. For ; *δ* = 1 and *δ*=2, *f*(*k*) is approximately (1 *+t +β*) *× A*, provided that *t + β* is sufficiently large. Similarly, other integral values of *r* yield closed form expressions, which provide some insight as to the behavior or the posterior expectation of *X.*

From Eqs. (3) and (11), the posterior predictive second moment for *X*, given that *X ⩾ A*, is

$$\int^{\left(2\right)}\left(k\right)=A^{2}\times E[\frac{\alpha }{\alpha -2}\left|D,X⩾A\right].$$ (17)

If *α* > 2 and the *α priori* distribution for *α* − 2 is of the Gamma form, with parameters *δ*,*β*, the posterior predictive variance for *X* will be finite, provided that *δ* >1. Closed form expressions can be obtained when *r* is a positive integer, just as with-the corresponding predictive first moment.

## 4. *k* < ∞

One of our purposes in this article is to show that prediction can be very sensitive to the *a priori* information introduced regarding *L*, and that it is essential to incorporate strong *a priori* information as to the magnitude of this quantity in order to obtain realistic forecasts. No closed form results are available apart from those of the last section. We consider now various *a priori* distributions for or. In the previous analysis it was not possible to give *α* a uniform distribution, since this would require *β* = 0 and *δ* = 1, in which case with infinite *k* the expectation is infinite. However, with a finite upper bound for *X*, we obtain a finite expectation for any *α* ≥ 0, and in fact even for negative *α*, although this case is of little interest.

Table 2 displays results for the case of a uniform *a priori* distribution for a, using a finite grid of possible values for *α* between LB = 1.001 and UB = 1.999, several values of *r* and *t*, and several choices of *L.* The prior expectation and standard deviation for α and 1.50 and .29, respectively. Table 3 gives such results for a uniform *a priori* distribution, using a finite grid of values between LB = .001 and UB = 1.999, in which case the prior expectation and standard deviation for *α* are 1.00 and .58, respectively. In these tables the column labelled “POST” gives the posterior expectation and standard deviation for *a*, the column labelled “PRED” gives the posterior predictive expectation and standard deviation for the next observation *X*, and the column labelled “DIST” gives the posterior probability that *X* is larger than 2,^−^5, and 10 times *A*, respectively.

So far we have only considered very strong *a priori* knowledge, such as in Table 1 where *α* is known, and very weak *a priori* knowledge, such as the uniform distributions of Tables 2 and 3. In applications it is important also to be able to input an *a priori* distribution for *α* in which some values are singled out as being given substantially more weight than others. A useful family of *a priori* distributions for *α* for this purpose is the three-parameter log-normal family. Suppose that ln(*α−γ*) ~ *N*(*μ,σ*^2^). This is the three-parameter log-normal distribution with threshold parameter *γ*, and is a very convenient and interesting family with which to make inference about *α.* See Aitchison and Brown [31], and Hill [32] for some properties of this distribution. The integrations in this case again have to be done by numerical analysis. In Table 4 we present results for the case *γ* = 1, with *α* taking values between LB = 1.001 and UB = 10. The prior mean and standard deviation for *α* are 1.50 and .61, respectively.

## 5. Discussion of Tables

If *α* > 2 then for fixed known *α* there is no problem with infinite first and second moments. This is also the case when *α* is unknown, except that the *a priori* distribution for *α* must give sufficiently small weight to values near 2 in order that the second moment be finite. However, the case *α* > 2, although of some interest, does not deal with truly long-tailed distributions. For *α* > 1, and using a Gamma prior distribution for *α* − 1 with *δ* > 1, as *k→∞* the posterior moments of *X* converge to the limiting results discussed in Sec. 3, such as in Eq. (16). We observe, however, that the convergence is quite slow. For values of *k* in the practical range, say *L* ≤ 10, the results are not very sensitive to the precise value of *L*, but are quite different from the limiting results, because the convergence is so slow. For example, the theoretical value for the multiplier of *A* when *r* = 0,*t* = 1,*δ* = 2, *β* = 1, is 3. Using UB = 10, when *L* = 10^12^ the calculated value for this multiplier is 2.86, and it is still only 2.98 when *L* = 10^80^. For L ≤ 10^6^, however, the multiplier is less than 2.16, and for values *L* ≤ 10, it is at most 2. Thus even in this case, where the posterior expectation exists for *k* = ∞, it can still be important to use a realistic value for *L.* Although this case can be described as a genuine long-tailed distribution, in order for the posterior expectation of *X* to be finite when *k* = ∞, it is necessary to take *δ* > 1, and so the *a priori* expectation for *α* must be larger than 1 +1/*β*.

A case of substantial practical importance is that in which the *a priori* information about a is weak, apart from the knowledge that 1 *< α <2.* There is substantial empirical data on incomes, stock-market prices, city sizes, the distribution of biological genera and species, and many other variables, for which *α ≤* 2. See Yule [15] and Zipf [14]. However, there is no known theoretical reason for taking the *a prion* distribution of *α* to be of the Gamma form, or for taking δ > 1. In the case of weak *a priori* information, the likelihood function is approximately proportional to the posterior density for *α.* See the stable estimation argument of Savage [33] and Edwards, Lindman and Savage [34]. For either classical statisticians, to whom the *a priori* distribution rs non-existent or “unknown,” or to Bayesians who prefer to use some form of “uninformative” prior distribution, the results of Table 2 should be quite reassuring. It is possible, despite the delicacy at co to obtain robust answers. It may be noted in this table that typically the posterior predictive expectation of *X_n_*_+1_, given that it is between. *X*_(1)_ and 10×*x*_(1)_, is some modest multiple of the largest observation, at most 3 ×*x*_(1)_; and it is at most 5 × *x*_(1)_ when *L ≤* 100 100. This is as it should be. One does not, for example, anticipate wind strengths that are some enormous factor times the largest yet experienced, even given that we set a new record wind strength. By comparing Table 1 for *α* = 1.50 known, with Table 2 for the case *r* = 3, *t* = 2, we see that there is little sensitivity in either the predictive moments or the predictive probabilities. For example, when *L* =5, Table 1 gives predictive moments of 1.82 and .87, and predictive probabilities of .35, .09, and .03; while Table 2 gives predictive moments of 1.83 and .88, and predictive probabilities of .36, .10, and .04. The greatest discrepancies occur for very large values of *L*, such as 10^6^, which are inappropriate for most real-world applications.

Another case of substantial interest is that in which *α* is uniform from 0 to 2, so that even more extreme long-tailed behavior is possible. Again results are not very sensitive to the choice of *a priori* distribution, provided that *L* is not too large. For example, Table 3 with r = 3,*t*= 2,*L* = 5, gives the predictive moments as 1.90 and .93, and the predictive probabilities as .42, .15, and .08. Although there is a real change from the results of Tables 1 and 2, it is of limited extent, and is in the direction of making the predictive distribution longer-tailed, as was to be expected. If anything, one might be surprised that allowing *α* to get close to 0, as with this *a priori* distribution, did not move the predictive distribution much further to the right.

The final case of great interest is where some definite *a priori* information is input, as we do here with the log-normal distribution. Table 4, for the case *γ* = 1,*r* = 3,*t* = 2,*L* = 5, gives 1.86 and .90 as predictive moments, and .39, .12, and .05, as predictive probabilities. These results are close to those of Table 2, in which *α* has the same *a priori* expectation as in Table 4.

The reader may compare these various tables for other values of the parameters, to examine the effect of long-tailed sample data, greater sample sizes, cases where the *a priori* information is less concordant with the data, and the effect of *L.* For example, in Table 3 with *r* = 2,*t* = 3, so that *â* = .67, and *L* = 5, the predictive moments are 2.07 and 1.01, while the predictive probabilities are .56, .29, and .19. Again, provided that a realistic upper bound for *L* is chosen, such as 10, the changes from previous values are real but of limited magnitude, and in the direction to be anticipated.

Armed with this information, let us now examine real-world data on city sizes. Table 5 gives the sizes of the 30 largest cities in the United States in 1940 and 1988. They are first presented in descending order, and then in a randomly chosen permutation. The data for 1940 was previously analysed in [1] to illustrate use of the tail-index method. The upper tail of such city size data is generally regarded as being modelled by Zipf’s law, with some tail-index *α.* Tables 6 and 7 give the running forecasts, and their standard deviations, for the next observation, based upon the permutation. We imagine, in other words, that a random sample has been taken from the population, and that we successively forecast the magnitude of each upcoming record value. In this way we simulate the actual forecasting of future record values based upon a random sample from a population. It is well known that sampling (with or without replacement) from a finite population generates an exchangeable sequence. Because our forecast of the magnitude of the next record value depends only upon the upper order statistics of the past data, and not directly upon how many past values have been observed, we put forth the same expectation for the magnitude of the next record value, until we observe a new record value.

The record values (with the first value taken as a record value by default) for Table 6 occurred at times 1, 5, 21, and had the values 1931, 3397, 7455, respectively. Table 6 gives the 1940 forecasts for *L* = 3.5,10, where each forecast is based upon all the past data up to the time of the forecast, and uses only the current upper two order statistics of the data, so *r* = 1. The column labelled $\hat{\alpha }$ gives the current maximum-likelihood estimate of *a* based upon the two upper order statistics, so $t=\frac{1}{\alpha }$. The first row of Table 6 would be read as follows. Based upon the two largest order statistics (1931, 859) at lime *2* in the 1940 permuted sequence, the estimate of *α* is 1.235. This data (with *r* = 1 and *t* = .810) is used to obtain the posterior distribution for *α*, for ;t uniform *a priori* distribution on the interval from 0 to 2. Forecasts and standard deviations are then presented for *L* =3,5,10. For example the *L* = 3 forecast of the next record value is 3146 with *a* standard deviation of 1023, this forecast being made using only the previous records of 1931 and 859. The realized value turned out to be 3397. Note that most of the actual values are well within 1 standard deviation of the forecast. The row ‘?’ forecasts a next record value, based upon all the past data, as though the population were not complete, and is given only for illustrative purposes. Table 7 repeats the analysis for the 1988 city size data. The record values occurred at trials 1, 4, 7, 14, and had the values 987, 1647, 2978, 7353, respectively.

This type of forecasting problem, based upon a random sample from a fixed population, is used to illustrate the procedure in connection with an exchangeable sequence of observations. As shown by de Finetti, and discussed in [35], one can always represent real-world exchangeable sequences in terms of limits arising in sampling from a finite population. The exchangeable case is the simplest scenario in which our methods can be usefully applied. More generally, one must deal with evolutionary processes, as for example when successive records are set over time. For example, if we consider the successive Olympic High Jump records, since 1880, we must keep in mind that we are not sampling from a fixed population, and that changes in technique and general level of physical fitness over time, may have a substantial effect. Similarly, in considering the next record value of some stock market index, such as the Dow Jones, there may be time trends that must be taken into account. However, even in such examples as these, local exchangeability over sufficiently short time periods may be a reasonable assumption, and appropriate modification of the basic forecasting procedure proposed in this article can be developed.

## 6. Conclusions

We believe that the above studies indicate that it is possible to make effective inference and predictions about record values. Our methodology can be used both with uniform *a priori* distributions, such as represented in Tables 2 and 3, and with more informative *a priori* distributions such as in Table 4. The case that is perhaps of greatest interest for applications is that of the three-parameter log-normal distribution with threshold taken to be 1 or 0, as may seem appropriate. Uniform *a priori* distributions can, for practical purposes, be represented as special cases of such log-normal distributions. We believe that it is important to study sensitivity of results to choice of *a priori* distribution, as recommended in (36,30], The choice of rand of *L* can be implemented by Baycsian data-analytic techniques, such as described in [1,25]. Here in our forecast of city sizes we took *r* = 1, but substantial improvements could result from a Bayesian decision-theoretic choice of *r.*

There are some basic issues concerning the use of finite models, versus infinite idealized models, that are especially pertinent in connection with the problem of prediction for long-tailed distributions. If one took the conventional idealized model literally in our example, then the analysis of Secs. 1 and 2 demonstrates that there arc some logical difficulties, if one also views the observations as unbounded. For in the case of greatest interest, where it is known that is 1 ≤ *α* ≤ 2, the posterior first moment may be infinite, even though it is plainly unreasonable to make a prediction of more than ft few multiples of the largest observation yet seen. The issue is resolved here by treating the algebraic model for the tail as only an approximation, valid in some finite domain. In this case the algebraic tail is compatible with both the data, and with putting forth sensible predictions for squared error lots. See [24] for discussion of the finite/infinite question in connection with Stcinian shrinkage estimators.

The issue regarding infinite predictive moments thus turns out to be largely irrelevant for forecasting, provided that one is comfortable with using some reasonable upper bound for the observable variables. Careless use of infinite models, ignoring the fact that realistic finite upper bounds are usually available, might instead have led one to the conclusion that theory-based forecasting is impossible in the case *α* ≤ 2. Since all statistical analyses must eventually be done on a computer with finite memory, such infinite models are at best only useful guides, and their careless use can lead to numerous apparent paradoxes, which have no real-world importance. The primary conclusion of this article is that provided that a finite upper bound for the observations can be supplied, as is ordinarily the case, it is possible to make effective predictions of future record values. The forecasts that we have obtained, employing such finite upper bounds, are by no means perfect, but they do at least put one in the right ballpark, with predictions that are at most a few multiples of the previous record value. I am not aware of other methods available at present that do so.

Forecasting is always difficult, and perhaps even more so for the case of record values in the case of long-tailed distributions. Nonetheless, often such forecasts are important in the decision-making process, and must somehow or other be put forth. We have suggested a Bayesian methodology which can make systematic use both of *a priori* information and of the current data. When used with care, we believe these methods can be of value in a variety of areas.

## Figures and Tables

**Table 1:** Fixed ALPHA

ALPHA	FRED		DIST			BOUND

*α*	E^*^(*X*)	SD^*^(*X*)	2	3	5	*L*
.10	1.12	.07	.93	.85	.79	1.25
.10	1.23	.14	.93	.85	.79	1.50
.10	1.44	.29	.93	.85	.79	2
.10	1.80	.57	.93	.85	.79	3
.10	2.43	1.12	.93	.85	.79	5
.10	3.75	2.45	.93	.85	.79	10
.10	18.70	23.46	.93	.85	.79	100
.10	734.88	1715.25	.93	.85	.79	10^4^
.10	37297.27	1.28 ×10^5^	.93	.85	.79	10^6^
.50	1.12	.07	.71	.45	.32	1.25
.50	1.22	.14	.71	.45	.32	1.50
.50	1.41	.28	.71	.45	.32	2
.50	1.73	.56	.71	.45	.32	3
.50	2.24	1.07	.71	.45	.32	5
.50	3.16	2.22	.71	.45	.32	10
.50	10.00	16.43	.71	.45	.32	100
.50	100.05	571.77	.71	.45	.32	10^4^
.50	3001.62	18257.56	.71	.45	.32	10^6^
.90	1.12	.07	.54	.23	.13	1.25
.90	1.22	.14	.54	.23	.13	1.50
.90	1.39	.28	.54	.23	.13	2
.90	1.66	.54	.54	.23	.13	3
.90	2.05	1.00	.54	.23	.13	5
.90	2.67	1.93	.54	.25	.13	10
.90	5.35	10.12	.54	.23	.13	100
.90	13.62	142.79	.54	.23	.13	10^4^
.90	26.86	1806.66	.54	.23	.13	10^6^
1.10	1.12	.07	.47	.17	.08	1.25
1.10	1.21	.14	.47	.17	.08	1.50
1.10	1.38	.28	.47	.17	.08	2
1.10	1.63	.53	.47	.17	.08	3
1.10	1.97	.96	.47	.17	.08	5
1.10	2.46	1.78	.47	.17	.08	10
1.10	4.09	7.73	.4^−1^	.17	.08	100
1.10	6.62	69.46	.47	.17	.08	10^4^
1.10	8.24	554.67	.47	.17	.08	10*
1.50	1.11	.07	.35	.09	.03	1.25
1.50	1.21	.14	.35	.09	.03	1.50
1.50	1.36	.27	.35	.09	.03	2
1.50	1.57	.50	.35	.09	.03	3
1.50	1.82	.87	.35	.09	.03	5
1.50	2.12	1.49	.35	.09	.03	10
1.50	2.70	4.44	.35	.09	.03	100
1.50	2.97	16.98	.35	.09	.03	10^4^
1.50	3.00	54.72	.35	.09	.03	10^6^
1.90	1.11	.07	.27	.05	.01	1.25
1.90	1.20	.14	.27	.05	.01	1.50
1.90	134	.27	.27	.05	.01	2
1.90	1.51	.48	.27	.05	.01	3
1.90	1.69	.78	.27	.05	.01	5
1.90	1.87	1.22	.27	.05	.01	10
1.90	2.08	2.61	.27	.05	.01	100
1.90	2.11	4.93	.27	.05	.01	10^4^
1.90	2.11	7.23	.27	.05	.01	10^6^

**Table 2:** Uniform prior, LB= 1.001, UB= 1.999, prior mean= 1.50, SD = .29

DATA		POST		PRED		DIST			BOUND

*r*	*t*	E*(*α*)	SD*(*α*)	E*(*X*)	SD*(*X*)	2	5	10	*L*
1	1	1.47	.29	1.11	.07	.37	.10	.04	1.25
1	1	1.47	.29	1.21	.14	.37	.10	.04	1.50
1	1	1.47	.29	1.36	.37	.27	.10	.04	2
1	1	1.47	.29	1.58	.51	.27	.10	.04	3
1	1	1.47	.29	1.84	.88	.37	.10	.04	5
1	1	1.47	.29	2.16	1.54	.37	.10	.04	10
1	1	1.47	.29	2.96	5.37	.37	.10	.04	10^2^
1	1	1.47	.29	3.82	38.95	.37	.10	.04	10^4^
1	1	1.47	.29	4.30	305.19	.37	.10	.04	10^6^
3	2	1.50	.28	1.11	.07	.36	.10	.04	1.25
3	2	1.50	.28	1.21	.14	.36	.10	.04	1.50
3	2	1.50	.28	1.36	.27	.36	.10	.04	2
3	2	1.50	.28	1.57	.51	.36	.10	.04	3
3	2	1.50	.28	1.83	.88	.36	.10	.04	5
3	2	1.50	.28	2.14	1.52	.36	.10	.04	10
3	2	1.50	.28	2.88	5.16	.36	.10	.04	10^3^
3	2	1.50	.28	3.63	35.99	.36	.10	.04	10^4^
3	2	1.50	.28	4.03	276.83	.36	.10	.04	10^5^
2	3	1.37	.27	1.11	.07	.39	.12	.05	1.25
2	3	1.37	.27	1.21	.14	.39	.12	.05	1.50
2	3	1.37	.27	1.37	.28	.39	.12	.05	2
2	3	1.37	.27	1.59	.51	.39	.12	.05	3
2	3	1.37	.27	1.87	.90	.39	.12	.05	5
2	3	1.37	.27	2.24	1.61	.39	.36	.36	10
2	2	1.37	.27	3.22	5.99	.39	.36	.36	10^2^
2	3	1.37	.27	4.43	46.85	.39	.36	.36	10^4^
2	3	1.37	.27	5.16	377.37	.39	.36	.36	10^6^
5	1	1.67	.25	1.11	07	.32	.07	.03	1.25
5	1	1.67	.25	1.21	.14	.32	.07	.03	1.50
5	1	1.67	.25	1.35	.27	.32	.07	.03	2
5	1	1.67	.25	1.55	.49	.32	.07	.03	3
5	1	1.67	.25	1.77	.84	.32	.07	.03	5
5	1	1.67	.25	2.02	1.40	.32	.07	.03	10
5	1	1.67	.25	2.49	4.05	.32	.07	.03	10^2^
5	1	1.67	.25	2.80	21.99	.32	.07	.03	10^4^
5	1	1.67	.25	2.93	152.19	.32	.07	.03	10^5^
1	5	1.22	.20	1.11	.07	.43	.15	.07	1.25
1	5	1.22	.20	1.21	.14	.43	.15	.07	1.50
1	5	1.22	.20	1.37	.28	.43	.15	.07	2
1	5	1.22	.20	1.61	.52	.43	.15	.07	3
1	5	1.22	.20	1.93	.93	.43	.15	.07	5
1	5	1.22	.20	2.36	1.71	.43	.15	.07	10
1	5	1.22	.20	3.68	6.97	.43	.15	.07	10^2^
1	5	1.22	.20	5.61	60.75	.43	.15	.07	10^4^
1	5	1.22	.20	6.94	512.81	.43	.15	.07	10^6^
30	20	1.52	.23	1.11	.07	.35	.09	.03	1.25
30	20	1.52	.23	1.21	.14	.35	.09	.03	1.50
30	20	1.52	.23	1.36	.27	.35	.09	.03	2
30	20	1.52	.23	1.57	.50	.35	.09	.03	3
30	20	1.52	.23	1.82	.87	.35	.09	.03	5
30	20	1.52	.23	2.12	1.50	.35	.09	.03	10
30	20	1.52	.23	2.78	4.81	.35	.09	.03	100
30	20	1.52	.23	3.29	28.02	.35	.09	.03	10^4^
30	20	1.52	.23	3.48	185.91	35	.09	.03	10^6^
20	30	1.08	.07	1.12	.07	.47	.18	.08	1.25
20	30	1.08	.07	1.22	.14	.47	.18	.08	1.50
20	31	1.08	.07	1.38	.28	.47	.18	.08	2
20	30	1.08	.07	1.63	.53	.47	.18	.08	3
20	30	1.08	.07	1.98	.96	.47	.18	.08	5
20	30	1.08	.07	2.48	1.80	.47	.18	.08	10
20	30	1.08	.07	4.21	8.01	.47	.18	.08	100
20	30	1.08	.07	7.27	78.63	.47	.18	.08	10^4^
20	30	1.08	.07	9.71	706.23	.47	.18	.08	10^6^
300	200	1.50	.09	1.11	.07	.35	.09	.03	1.25
300	200	1.50	.09	1.21	.14	.35	.09	.03	1.50
300	200	1.50	.09	1.36	.27	.35	.09	.03	2
300	200	1.50	.09	1.57	.50	.35	.09	.03	3
300	200	1.50	.09	1.82	.87	.35	.09	.03	5
300	200	1.50	.09	2.12	1.49	.35	.09	.03	10
300	200	1.50	.09	2.71	4.48	.35	.09	.03	10^2^
300	200	1.50	.09	3.00	18.24	.35	.09	.03	10^4^
300	200	.50	.09	3.04	67.25	.35	.09	.03	10^6^
200	300	1.01	.01	1.12	.07	.50	.20	.10	1.25
200	300	1.01	.01	1.22	.14	.50	.20	.10	1.50
200	300	1.01	.01	1.39	.28	.50	.20	.10	2
200	300	1.01	.01	1.65	.53	.50	.20	.10	3
200	300	1.01	.01	2.01	.97	.50	.20	.10	5
200	300	1.01	.01	2.55	1.85	.50	.20	.10	10
200	300	1.01	.01	4.59	8.73	.50	.20	.10	10^2^
200	300	1.01	.01	8.88	95.94	.50	.20	.10	10^4^
200	300	1.01	.01	13.01	941.80	.50	.20	.10	10^6^

**Table 3:** Uniform prior, LB = 0.001, UB=1.999, prior mean = 1.00, SD = 58

DATA		POST		PRED		DIST			BOUND

*r*	*t*	E*(*α*)	SD(*α*)	E*(*X*)	SD*(*X*)	2	5	10	*L*
1	1	1.09	.51	1.12	.07	.50	.24	.15	1.25
1	1	1.09	.51	1.21	.14	.50	.24	.15	1.50
1	1	1.09	.51	1.38	.28	.50	.24	.15	2
1	1	1.09	.51	1.64	.53	.50	.24	.15	3
1	1	1.09	.51	1.99	.98	.50	24	.15	5
1	1	1.09	.51	2.55	1.90	.50	.24	.15	10
1	1	1.09	.51	5.71	11.70	.50	.24	.15	100
1	1	1.09	.51	59.13	474.46	.50	.24	.15	10^4^
1	1	1.00	.51	1599.34	26459.07	.50	.24	.15	10^5^
3	2	1.31	.42	1.11	.07	.42	.15	.08	1.25
3	2	1.31	.42	1.21	.14	.42	.15	.08	1.50
3	2	1.31	.42	1.37	.28	.42	.15	.08	2
3	2	1.31	.42	1.60	.32	.42	.15	.08	3
3	2	1.31	.42	1.90	.93	.42	.15	.08	5
3	2	1.31	.42	2.32	1.70	.42	.15	.08	10
3	2	1.31	.42	3.98	8.23	.42	.15	.08	100
3	2	1.31	.42	15.97	209.46	.42	.15	.08	10^4^
3	2	1.31	.42	187.75	8561.51	.42	.15	.08	10^6^
2	3	.90	.44	1.12	.07	.56	.29	.19	1.25
2	3	.90	.44	1.22	.14	.56	.29	.19	1.30
2	3	.90	.44	1.39	.28	.56	.29	.19	2
2	3	.90	.44	1.67	.54	.56	.29	.19	3
2	3	.90	.44	2.07	1.01	.56	.29	.19	5
2	3	.90	.44	2.72	2.01	.56	.29	.19	10
2	3	.90	.44	6.76	13.07	.56	.29	.19	100
2	3	.90	.44	70.77	511.32	.56	.29	.19	10^4^
2	3	.90	.44	1619.79	26176.42	.56	.29	.19	16^6^
5	1	1.64	.29	1.11	.07	.33	.08	.03	1.25
5	1	1.64	.29	1.21	.14	.33	.08	.03	1.50
5	1	1.64	.29	1.35	.27	.33	,08	.03	2
5	1	1.64	.29	1.55	.50	.33	.08	.03	3
5	1	1.64	.29	1.78	.85	.33	.08	.03	5
5	1	1.64	.29	2.04	1.43	.33	.08	.03	10
5	1	1.64	.29	2.62	4.57	.33	.08	.03	100
5	1	1.64	29	3.57	50.32	.33	.08	.03	10^4^
5	1	1.64	.29	7.89	1247.04	.33	.08	.03	10^6^
1	5	.40	.28	1.12	.07	.77	.57	.47	1.25
1	5	.40	.28	1.23	.14	.77	.57	.47	1.50
1	5	.40	.28	1.42	.29	.77	.37	.47	2
1	5	.40	.28	1.75	.56	.77	.37	.47	3
1	5	.40	.28	2.29	1.09	.77	.37	.47	5
1	5	.40	.28	3.33	2.31	.77	.57	.47	10
1	5	.40	.28	12.74	19.46	.77	.37	.47	100
1	5	.40	.28	309.24	1125.56	.77	.37	.47	10^4^
1	5	.40	.28	11841.58	72682.37	.77	37	.47	10^6^
30	20	1.51	.24	1.11	.07	.36	.09	.04	1.25
30	20	1.51	.24	1.21	.14	.36	.09	.04	1.50
30	20	1.51	.24	1.36	.27	.36	.09	.04	2
30	20	1.51	.24	1.57	.50	.36	.09	.04	3
30	20	1.51	.24	1.82	.87	.36	.09	.04	5
30	20	1.51	.24	2.13	1.50	.36	.09	.04	10
30	20	1.51	.24	2.81	4.92	.36	.09	.04	100
30	20	1.51	.24	3.42	32.25	.36	.09	.04	10^4^
30	20	1.51	.24	3.80	284.87	.36	.09	.04	10^6^
20	30	.70	.15	1.12	.07	.62	.33	.21	1.25
20	30	.70	.15	1.22	.14	.62	33	.21	1.50
20	30	.70	.15	1.40	.28	.62	.33	.21	2
20	30	.70	.15	1.70	.55	.62	.33	.21	3
20	30	.70	.15	2.14	1.03	.62	33	.21	5
20	30	.70	.15	2.91	2.09	.62	.33	.21	10
20	30	.70	.15	7.49	13.50	.62	.33	.21	100
20	30	.70	.15	46.87	366.86	.62	.33	.21	10^4^
20	30	.70	.15	357.77	10632.53	.62	.33	.21	10^6^
300	200	1.50	.09	1.11	.07	.35	.09	.03	1.25
300	200	1.50	.09	1.21	.14	.35	.09	.03	1.50
300	200	1.50	.09	1.36	.27	.35	.09	.03	2
300	200	1.50	.09	1.57	.50	.35	.09	.03	3
300	200	1.50	.09	1.82	.87	.35	.09	.03	5
300	200	1.50	.09	2.12	1.49	.35	.09	.03	10
300	200	1.50	.09	2.71	4.48	.35	.09	.03	100
300	200	1.50	.09	3.00	18.24	.35	.09	.03	10^4^
300	200	1.50	.09	3.04	67.25	.35	.09	.03	10^6^
200	300	.67	.05	1.12	.07	.63	.34	.22	1.25
200	300	.67	.05	1.22	.14	.63	.34	.22	1.50
200	300	.67	.05	1.40	.28	.63	.34	.22	2
200	300	.67	.05	1.70	.55	.63	.34	.22	3
200	300	.67	.05	2.16	1.04	.63	.34	.22	5
200	300	.67	.05	2.94	2.10	.63	.34	.22	10
200	300	.67	.05	7.62	13.58	.63	.34	.22	100
200	300	.67	.05	41.81	330.65	.63	.34	.22	10^4^
200	300	.67	.05	212.71	7448.90	.63	.34	.22	10^6^

**Table 4:** Log-normal prior, LB = 1.001, UB = 10, *γ* = 1, *µ* = −1.19, *σ* = 1, prior mean = 1.50, SD = .61

DATA		POST		PRED		DIST			BOUND

*r*	*t*	E*(*α*)	SD*(*α*)	E*(*X*)	SD*(*X*)	2	5	10	*L*
1	1	1.39	.38	1.11	.07	.39	.12	.05	1.25
1	1	1.39	.38	1.21	.14	.39	.12	.05	1.50
1	1	1.39	.38	1.37	.28	.39	.12	.05	2
1	1	1.39	.38	1.59	.51	.39	.12	.05	3
1	1	1.39	.38	1.87	.90	.39	.12	.05	5
1	1	1.39	.38	2.24	1.62	.39	.12	.05	10
1	1	1.39	.38	3.26	6.09	.39	.12	.05	100
1	1	1.39	.38	4.48	46.61	.39	.12	.05	10^4^
1	1	1.39	.38	5.15	353.95	.39	.12	.05	10^6^
3	2	1.41	.38	1.11	.07	.39	.12	.05	1.25
3	2	1.41	.38	1.21	.14	.39	.12	.05	1.50
3	2	1.41	.38	1.36	.27	.39	.12	.05	2
3	2	1.41	.38	1.59	.51	.39	.12	.05	3
3	2	1.41	.38	1.86	.90	.39	.12	.05	5
3	2	1.41	.38	2.22	1.60	.39	.12	.05	10
3	2	1.41	.38	3.18	5.92	.39	.12	.05	100
3	2	1.41	.38	4.30	44.35	.39	.12	.05	10^4^
3	2	1.41	.38	4.90	332.81	.39	.12	.05	10^6^
2	3	1.27	.23	1.11	.07	.42	.14	.06	1.25
2	3	1.27	.23	1.21	.14	.42	.14	.06	1.5
2	3	1.27	.23	1.37	.28	.42	.14	.06	2
2	3	1.27	.23	1.61	.52	.42	.14	.06	3
2	3	1.27	.23	1.91	.92	.42	.14	.06	*5*
2	3	1.27	.23	2.31	1.67	.42	.14	.06	10
2	3	1.27	.23	3.50	6.57	.42	.14	.06	100
2	3	1.27	.23	5.02	52.43	.42	.14	.06	10^4^
2	3	1.27	.23	5.02	52.43	.42	.14	.06	10^6^
5	1	2.34	1.17	1.11	.07	.25	.06	.02	1.25
5	1	2.34	1.17	1.20	.14	.25	.06	.02	1.50
5	1	2.34	1.17	1.32	.26	.25	.06	.02	2
5	1	2.34	1.17	1.48	.47	.25	.06	.02	3
5	1	2.34	1.17	1.65	.77	.25	.06	.02	5
5	1	2.34	1.17	1.83	1.26	.25	06	.02	10
5	1	2.34	1.17	2.22	3.82	.25	.06	.02	100
5	1	2.34	1.17	2.58	24.18	.25	.06	.02	10^4^
5	1	2.34	1.17	2.74	170.55	.25	.06	.02	10^6^
1	5	1.18	.14	1.11	.07	.44	.15	.07	1.25
1	5	1.18	.14	1.21	.14	.44	.15	.07	1.50
1	5	1.18	.14	1.38	.28	.44	.15	.07	2
1	5	1.18	.14	1.62	.52	.44	.15	.07	3
1	5	1.18	.14	1.94	.94	.44	.15	.07	5
1	5	1.18	.14	2.38	1.73	.44	.15	.07	10
1	5	1.18	.14	3.77	7.11	.44	.15	.07	100
1	5	1.18	.14	5.73	60.45	.44	.15	.07	10^4^
1	5	1.18	.14	6.95	483.26	.44	.15	.07	10^6^
30	20	1.40	.22	1.11	.07	.38	.11	.04	1.25
30	20	1.40	.22	1.21	.14	.38	.11	.04	1.50
30	20	1.40	.22	1.36	.27	.38	.11	.04	2
30	20	1.40	.22	1.58	.51	.38	.11	.04	3
30	20	1.40	.22	1.86	.90	.38	.11	.04	5
30	20	1.40	.22	2.21	1.58	.38	.11	.04	10
30	20	1.40	.22	3.09	5.58	.38	.11	.04	100
30	20	1.40	.22	3.92	36.97	.38	.11	.04	10^4^
30	20	1.40	.22	4.28	252 19	.38	.11	.04	10^6^
20	30	1.10	.07	1.11	.07	.47	.17	.08	1.25
20	30	1.10	.07	1.21	.14	.47	.17	.08	1.50
20	30	1.10	.07	1.38	.28	.47	.17	.08	2
20	30	1.10	.07	1.63	.53	.47	17	.08	3
20	30	1.10	.07	1.97	.96	.47	.17	.08	5
20	30	1.10	.07	2.46	1.78	.47	.17	.08	10
20	30	1.10	.07	4.09	7.76	.47	.17	.08	100
20	30	1.10	.07	6.75	7I.88	.47	.17	.08	10^4^
20	30	1.10	.07	8.61	607.81	.47	.17	.08	10^6^

**Table 5:** City size × 10^− 3^ data

1940		1988	
Ordered	Permuted	Ordered	Permuted
7455	1931	7353	987
3397	859	3353	727
1931	302.3	2978	532
1623	305	1698	1647
1504	3397	1647	522
878	368	1070	599
859	816	1036	2978
816	587	987	465
771	399	941	1036
672	1623	924	502
663	456	751	434
635	387	738	511
587	771	732	1070
576	635	727	7353
495	492	645	481
492	301.2	635	732
456	495	617	941
430	663	599	3353
399	306	578	578
387	878	570	570
385	7455	532	1698
368	325	521	617
325	322	511	924
322	319	502	738
319	576	492	645
306	302.2	481	751
305	672	465	492
302.3	430	439	635
302.2	1504	434	427
301.2	385	427	439

**Table 6:** Forecast uf 1940 oily sizes × 10^−3^

City size	$\hat{\alpha }$	Forecast			Forecast SD		
		3	5	10	3	5	10
3397	1.235	3146	3810	4831	1023	1869	3596
7455	1.770	5500	6621	8295	1787	3244	6166
(?)	1.272	12137	14694	18608	3944	7209	13844

**Table 7:** Forecast of 1988 city sizes × 10^−3^

City size	$\hat{\alpha }$	Forecast			Forecast SD		
		3	5	10	3	5	to
1647	3.271	1588	1899	2351	516	929	1743
2978	1.953	2663	3202	4001	865	1568	2973
7353	1.688	4824	5810	7290	1569	2847	5420
(?)	1.106	17.007	14581	18574	3904	7154	13824

## Footnotes

1 For those unfamiliar with exchangeability, il may be remarked that exchangeable sequences are strictly stationary processes and can be strongly dependent. An interesting and important class of exchangeable processes consists of the Markov-Pólya processes, discussed in [3,4,5,6], which play a major role in the theory of stochastic chaos.

2 Stime may think that because of such issues one should be considering inference about percentiles, such as the median, rather than the expectation. However, means are often of particular interest and importance in real-world problems, and of course are appropriale for squared error loss. If there were no technical difficulties at infinity with the expectation, would anyone argue against its use for prediction?

3 Instead of requiring that the mass be exactly 0 beyond a certain known bound *K*, one can alternatively require that the mass beyond this bound be so negligible as to be of no interest. In the subjective Bayesian approach it would be remarkable for anyone to have a probability of 0, to infinitely many decimal points, for a logically possible event. However, whether or not 0 is taken literally, in effect one ordinarily ignores values of the observation larger than the bound. For the purposes of this article we treat such negligible mass as though it were 0. An alternative and nearly equivalent way to deal with the problem is to consider only conditional inference, given that the observations arc no larger than the bound. A general theory and methodology for such conditional inference is proposed in [24].

4 See [30] for a general formulation of the robustness problem in Bayesian statistics.
